# Marginal Bone Level and Biomechanical Behavior of Titanium-Indexed Abutment Base of Conical Connection Used for Single Ceramic Crowns on Morse-Taper Implant: A Clinical Retrospective Study

**DOI:** 10.3390/jfb14030128

**Published:** 2023-02-26

**Authors:** Sergio Alexandre Gehrke, Antonio Scarano, Guillermo Castro Cortellari, Gustavo Vicentis Oliveira Fernandes, Alfredo Mikail Melo Mesquita, Marco Aurélio Bianchini

**Affiliations:** 1 Department of Research, Bioface/PgO/UCAM, Calle Cuareim 1483, Montevideo 11100, Uruguay; 2 Instituto de Bioingenieria, Universidad Miguel Hernández, Avda. Ferrocarril s/n., 03202 Elche, Spain; 3 Department of Biotechnology, Universidad Católica de Murcia (UCAM), 30107 Murcia, Spain; 4 Department of Materials Engineering, Pontificia Universidade Católica do Rio Grande do Sul, Porto Alegre 90619-900, Brazil; 5 Department of Innovative Technologies in Medicine & Dentistry, University of Chieti-Pescara, 66100 Chieti, Italy; 6 Periodontics and Oral Medicine Department, University of Michigan School of Dentistry, Ann Arbor, MI 48109, USA; 7 Department of Implantology, Paulista University (UNIP), São Paulo 01311-000, Brazil; 8 Post-Graduate Program in Implant Dentistry (PPGO), Federal University of Santa Catarina (UFSC), Florianópolis 88040-900, Brazil

**Keywords:** abutment, dental implant, rehabilitation, retrospective study, single ceramic crown

## Abstract

The goal of this retrospective clinical study was to evaluate the behavior of Morse-taper indexed abutments by analyzing the marginal bone level (MBL) after at least 12 months of function. Patients rehabilitated with single ceramic crowns between May 2015 and December 2020 received single Morse-taper connection implants (DuoCone implant) with two-piece straight abutment baseT used for at least 12 months, presenting periapical radiograph immediately after crown installation were enrolled. The position of the rehabilitated tooth and arch (maxilla or mandible), crown installation period, implant dimensions, abutment transmucosal height, installation site (immediate implant placement or healed area), associated with bone regeneration, immediate provisionalization, and complications after installation of the final crown were analyzed. The initial and final MBL was evaluated by comparing the initial and final X-rays. The level of significance was α = 0.05. Seventy-five patients (49 women and 26 men) enrolled had a mean period of evaluation of 22.7 ± 6.2 months. Thirty-one implant-abutment (IA) sets had between 12–18 months, 34 between 19–24 months, and 44 between 25–33 months. Only one patient failed due to an abutment fracture after 25 months of function. Fifty-eight implants were placed in the maxilla (53.2%) and 51 in the mandible (46.8%). Seventy-four implants were installed in healed sites (67.9%), and 35 were in fresh socket sites (32.1%). Thirty-two out of these 35 implants placed in fresh sockets had the gap filled with bone graft particles. Twenty-six implants received immediate provisionalization. The average MBL was −0.67 ± 0.65 mm in mesial and −0.70 ± 0.63 mm in distal (*p* = 0.5072). The most important finding was the statistically significant difference comparing the values obtained for MBL between the abutments with different transmucosal height portions, which were better for abutments with heights greater than 2.5 mm. Regarding the abutments’ diameter, 58 had 3.5 mm (53.2%) and 51 had 4.5 mm (46.8%). There was no statistical difference between them, with the following means and standard deviation, respectively, −0.57 ± 0.53 mm (mesial) and −0.66 ± 0.50 mm (distal), and −0.78 ± 0.75 mm (mesial) and −0.746 ± 0.76 mm (distal). Regarding the implant dimensions, 24 implants were 3.5 mm (22%), and 85 implants (78%) had 4.0 mm. In length, 51 implants had 9 mm (46.8%), 25 had 11 mm (22.9%), and 33 implants were 13 mm (30.3%). There was no statistical difference between the abutment diameters (*p* > 0.05). Within the limitations of this study, it was possible to conclude that better behavior and lesser marginal bone loss were observed when using abutment heights greater than 2.5 mm of transmucosal portion and when placed implants with 13 mm length. Furthermore, this type of abutment showed a little incidence of failures within the period analyzed in our study.

## 1. Introduction

Replacing missing teeth with osseointegrated implants has been highly approached, especially in recent decades. This type of treatment has become reliable and predictable, with satisfactory long-term results [1,2]. Maintenance of the peri-implant tissues’ health has been one of the main concerns and reasons for research development within the implant dentistry field. In addition, considering the systemic condition and local clinical characteristics of each patient, different factors of the implant system may influence the stability of tissues, such as the dimensions of the abutments connected to the implant, connection pattern, surface and chemical composition, and mainly, precision in the adaptation (fitting) between implant and abutment (IA) [3,4,5].

Regardless of the connection between IA, this fitting should favor load distribution and biological response and hinder bacterial proliferation [6,7,8]. IA interface has been referenced as the most significant factor for the longevity of dental implant rehabilitation treatment [9,10]. Moreover, different factors are related to the manufacture of implant components and their use, and/or clinical and laboratory indications can contribute to a misfit of this interface [8,11,12]. A misfit (microgap) is a microscopic space between the implant and the prosthetic abutment, and it can generate micromovements allowing the penetration of food, saliva, and microorganisms and their fluids [6,9,10,13]. The presence of bacteria in this area (IA interface) can generate inflammation of the peri-implant tissues and, consequently, bone loss, which may progress to peri-implantitis and/or even implant loss [11]. Furthermore, micromovements at the IA interface can cause tissue changes, generate wear of the parts by friction, loosen the fixation screw, or fracture the components [6,8,14].

Morse-taper connection implants have a precise tapered internal design. Through frictional retention, it promotes close adaptation between the overlapping surfaces of the IA, improving mechanical resistance and reducing microgap and rotational movements compared to other hexagonal connection systems [15,16,17]. In addition, the Morse-taper connection system reduced the tension points on the fixation screw and, consequently, the possibility of screw loosening, promoting better stability to the IA set [18,19,20]. Studies have also shown that Morse taper implants may present fewer complications to the peri-implant tissues and lower crestal bone loss [21,22,23].

Initially, all Morse-taper connection abutments manufactured and used were solid, made in one piece, without a passing screw. Thus, after the implant osseointegration period, the abutment was installed, torqued, and all the following procedures were performed directly on the abutment without removing it. Within this question, the ideal position of the implant was strictly necessary; otherwise, it may not always be clinically possible due to the anatomical conditions of the patients.

Owe to anatomical and/or mechanical limitations, new models of abutments have been required. Then currently, there are a wide variety of abutments available for Morse-taper connection implants [24]. In addition, for the fabrication of single crowns and/or bridges by the computer-aided design and computer-aided manufacturing (CAD/CAM) system, an intermediate titanium abutment as a base for the seating of these pieces is recommended [25,26]. Most of these abutments appeared in two pieces (an abutment and a passing screw). Also, because it is in two pieces, these abutments feature an index to determine their placement when attached to the implant, similar to an internal hexagon connection [27]. Studies have shown that the presence of the index on Morse-taper abutments can alter and/or prevent the frictional effect found on solid one-piece abutments [20,28,29]. On the other hand, with the development of abutments for Morse-taper with index, some facilities and simplifications to execute rehabilitation with implants were observed. In addition, it solved some adversities observed with conventional Morse-taper connections (without index).

However, in addition to the prefabricated titanium abutments, other abutments can be produced using different types of materials, such as zirconia or cobalt chromium. Zirconia abutments are usually manufactured by a CAD/CAM system, and cobalt-chromium abutments are cast in the laboratory. However, even though these ceramic materials have some advantages, such as better esthetics and adequate biocompatibility with peri-implant tissues, their mechanical behavior presents a higher incidence of fracture in ceramic abutments compared to titanium ones [30]. The inherent properties of ceramic materials, with lower resistance to fracture and less flexural strength than metals, may explain these findings [30,31]. Still, the risk of abutment fracture may also be affected by the material’s thickness, the implant’s position, and angulation concerning the final prosthetic restoration [30].

The abutments manufactured in cobalt-chromium can present both physical and chemical problems. The design and stability of the IA connection and the abutment material’s chemical composition and surface properties influence the function of implant-supported restorations and the adjacent soft tissue health and stability [32]. Jamari et al. [33] showed that titanium-on-titanium could bring advantages in biotribological, biocompatibility, and corrosion resistance. In a silicon study, these authors demonstrated that titanium-on-titanium has a superior ability to reduce contact pressure (more than 35%) than other connections using different metals. In addition, studies have shown that differences between metals (implant and abutment) can generate galvanic currents when in contact with saliva [34,35]. To avoid this phenomenon, it is prudent to follow the proposed recommendations, using different metals and always opting for the choice of the pairs titanium/titanium [36]. Another important point to be considered is the accuracy of adapting the abutments to the implant as a factor that can reduce mechanical and biological complications. Abutments manufactured in titanium by the same implant system (by numerical control lathe) have presented a superior adaptation than abutments manufactured by other methods [37,38].

Thus, considering the above-mentioned, titanium abutments as the basis of crowns using the CAD/CAM system seems to be the best clinical option. However, few clinical studies of the behavior of two-piece indexed abutments for Morse-taper implants are found in the literature [39]. Thus, the main objective of this retrospective clinical study was to evaluate the behavior of Morse-taper indexed abutments installed and in function for at least 12 months. The main hypothesis was that the indexed abutment could behave adequately to support individual crowns.

## 2. Materials and Methods

This study was approved by the Ethics Committee on Human Research of the UFSC (number 3,490,963-Florianopolis, Brazil) and followed the Declaration of Helsinki (1975, updated 2013). Furthermore, all patients received information about the nature of the study; they agreed and signed the informed consent authorizing data collection. One hundred and nine sets of IAs were installed to rehabilitate patients with single ceramic crowns between May 2015 and December 2020. All procedures were performed at the Center of Research in Dental Implants (CEPID) of the Health Sciences Center of the Federal University of Santa Catarina (UFSC, Florianopolis, Brazil).

### 2.1. Eligibility Criteria

**Figure 1:**
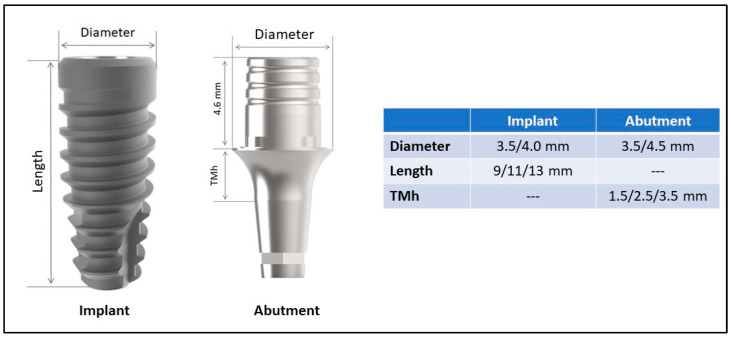
Representative image of the implant and abutment model with the dimensions considered in the present study. TMh = Transmucosal heigth.

It included (i) only patients who received single Morse-taper connection implants (DuoCone implant, Implacil De Bortoli, São Paulo, Brazil), (ii) rehabilitated with a two-piece straight abutment baseT (abutment and screw) (Implacil De Bortoli, São Paulo, Brazil), (iii) rehabilitated between May 2015 and December 2020, (iv) with at least 12 months in functional loading, (v) presenting periapical radiograph immediately after crown installation. The abutment dimensions used were 3.5 and 4.5 mm in diameter and 1.5, 2.5, and 3.5 mm in the transmucosal portion of height (TMh). This abutment is indicated as a support base when creating crowns using the CAD/CAM system. A representative image of the implant and abutment model with the dimensions considered in the present study is shown in Figure 1. Patients who (i) had an incomplete medical history or missing data, (ii) lost an implant and received another to substitute, (iii) had multiple and splinted crowns, and (iv) with any uncontrolled systemic condition were excluded from the study.

### 2.2. Data Collection and Variable Studied

Data related to the position of the rehabilitated tooth, arch (maxilla or mandible), crown installation period, implant dimensions, abutment transmucosal height, installation site conditions (immediate implant placement after tooth post-extraction or healed site), implant placement associated with bone regeneration or not and, immediate provisionalization or not. In all the cases analyzed, the implants were reopened after 3 months of installation, as per the general rule for these cases. The definitive crowns were installed an average of 5 months after implant placement. In addition, complications after installation of the final crown were analyzed, such as loosening the crown and/or fracture of the abutment/screw.

**Figure 2:**
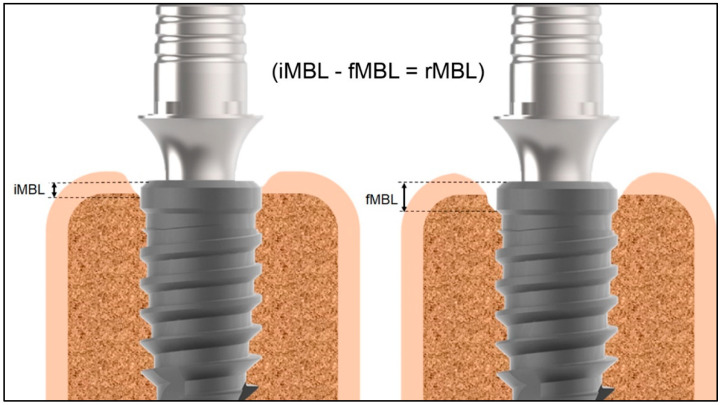
Schematic image of the measurement positions and the calculus used. iMBL = initial marginal bone level (MBL); fMBL = final MBL; and rMBL = result MBL.

For all patients included in the present study, a periapical radiograph was obtained using the parallel cone technique with a Rinn alignment system (Insight Film Kodak, Carestream, Rochester, NY, USA) and a digital rigid film-object X-ray source coupled to a beam-aiming device, to achieve reproducible exposure geometry. Then, at the time of the appointment, the following measurements were obtained: (i) initial marginal bone level (iMBL), which was measured from the implant platform to the most apical portion of the mesial and distal bone crest in the immediate radiographs after installation of the definitive crown; (ii) final marginal bone level (fMBL) that was measured following the same references on a radiograph as current as possible. Thereby, the difference between the iMBL and fMBL was calculated (fMBL-iMBL) and analyzed (rMBL). Figure 2 schematically shows the position considered for the measurements.

**Figure 3:**
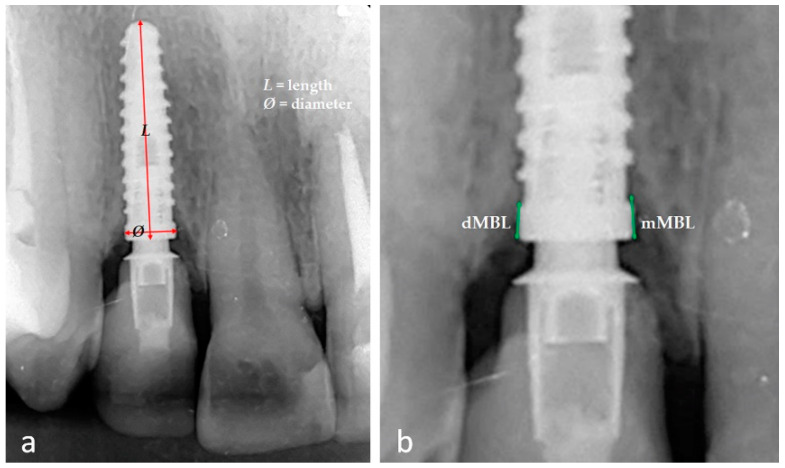
Representative image of the calibration using the implant diameter and length (**a**) and the measurement positions in mesial (mMBL) and distal (dMBL) (**b**).

All radiographic images were analyzed using the ImageJ software (National Institute of Health, Bethesda, MD, USA). For calibration before the measurements, the implant dimension (diameter and length) (Figure 3a), reported in the clinical history of each patient, was used as a reference value to adjust for any distortion. Each radiograph image was measured on a medical screen with a resolution of 1920 × 1080 and 10× magnification (Surgical Display Monitors-Medical Imaging Displays, Sony Inc., Tokyo, Japan). The marginal bone level was measured on immediate periapical radiography after installing the definitive crown (baseline) and follow-up. The segment between the implant neck and the first bone-to-implant contact was calculated and considered as both the mesial and distal position for each implant (Figure 3b). The same examiner makes all the measurements with much experience in dental implants and image analysis. Each measurement was repeated 3 times in each position (mesial and distal). These same measurements were repeated after 2 weeks to calculate an average used as reference values and the estimated error margin. The calculated intra-examiner error was, on average 0.05 mm, indicating that the intra-operator error was not statistically significant (*p* = 0.18 with 95% CI).

### 2.3. Statistical Analysis

After analyzing the data using the Kolmogorov-Smirnov test, it was verified Levene’s homogeneity of variance test for all data acquired. For bivariate analysis, Mann-Whitney U and Students-t tests were applied. Repeated-measures ANOVA was used to analyze the reduction in marginal bone loss. All comparison analysis was performed using GraphPad Prism 8 software (GraphPad Software, San Diego, CA, USA). The level of significance was set at α = 0.05.

## 3. Results

**Figure 4:**
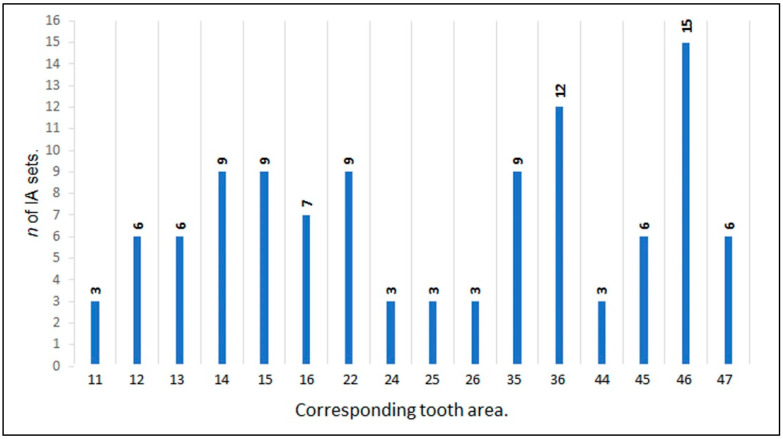
Distribution and number (*n*) of IA sets per corresponding tooth area.

Seventy-five patients were enrolled (49 women and 26 men), with a mean age of 59.3 years (ranging between 25 to 69 years), who received single ceramic crowns. A total of 109 IA sets were analyzed. The mean and standard deviation period of evaluation was 22.7 ± 6.2 months, distributed as follows: 31 IA sets (28.4%) between 12–18 months, 34 IA sets (31.2%) between 19–24 months, and 44 IA sets (40.4%) between 25–33 months. Only one patient failed due to an abutment fracture after 25 months of function. However, as this patient had adequate radiographic control, he was not excluded from the study. The distribution of implants by placement site is graphically presented in Figure 4.

Fifty-eight implants were placed in maxilla sites (53.2%), and 51 were installed in mandible sites (46.8%). Seventy-four implants were installed in healed sites (67.9%), and 35 were installed in fresh socket sites (32.1%). Thirty-two out of these 35 implants placed in fresh sockets had the gap filled with bone graft particles. ExtraGraft XG13 (Implacil, São Paulo, Brazil) was used in all cases. However, the quantity of material used, and the size of the defects was not adequately clarified in the patient records. Twenty-six implants received immediate provisionalization. Table 1 shows the mesial and distal MBL analysis between the different proposed variables and the statistical comparison.

**Figure 5:**
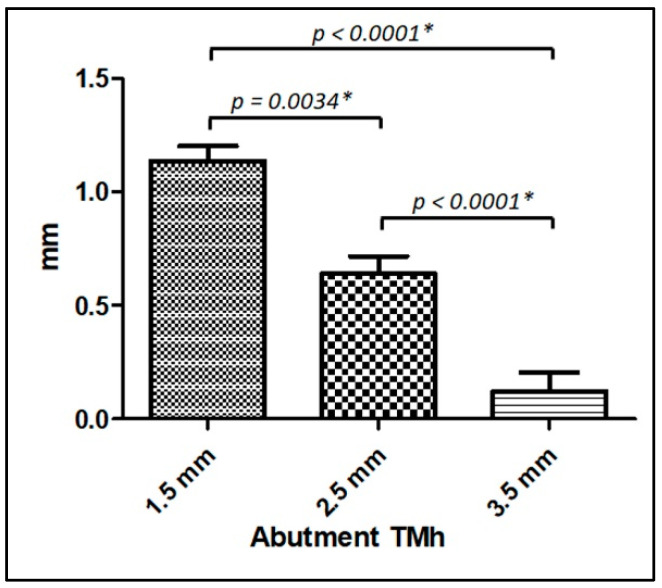
Graph of the average data obtained in each abutment model. TMh = transmucosal height; mm = millimeter; * Statistically significant difference.

The average rMBL was −0.67 ± 0.65 mm in mesial and −0.70 ± 0.63 mm in distal, without a statistical difference (*p* = 0.5072). The most important finding was the statistically significant difference comparing the values obtained for rMBL between the abutments with different transmucosal height portions. The rMBL values were better for abutments with heights greater than 2.5 mm. Table 2 shows the data of the abutment quantity of each transmucosal height used, the rMBL calculated, and the statistical difference between them. Figure 5 graphically presents the overall means (between mesial and distal rMBL) of each abutment model and their statistical comparison.

**Figure 6:**
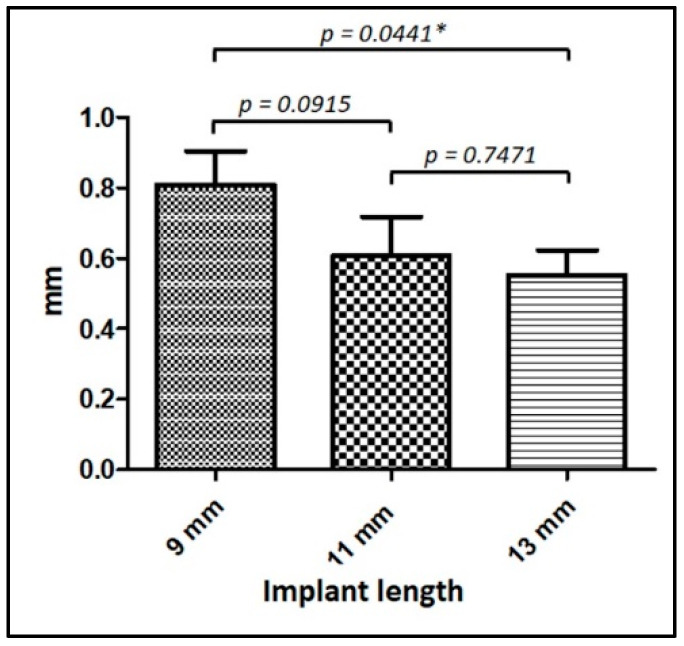
Graphic of the average data obtained for each implant length and the statistical difference between them for the rMBL. mm = millimeter; * Statistically significant difference.

Regarding the abutments’ diameter, 58 had 3.5 mm (53.2%), and 51 had 4.5 mm (46.8%). There was no statistical difference between them, with the following means and standard deviation, respectively, −0.57 ± 0.53 mm (mesial) and −0.66 ± 0.50 mm (distal), and −0.78 ± 0.75 mm (mesial) and −0.746 ± 0.76 mm (distal). Regarding the implant dimensions, 24 implants were 3.5 mm (22%), and 85 implants (78%) had 4.0 mm. In length, 51 implants had 9 mm (46.8%), 25 had 11 mm (22.9%), and 33 implants were 13 mm (30.3%). Table 3 shows the data comparison of the implant dimensions (diameter and length). Figure 6 shows the overall means (between mesial and distal rMBL) of each implant length and the statistical comparison between them graphically. We can observe that implants of 13 mm in length have less marginal bone remodeling.

## 4. Discussion

The present study aimed to evaluate the behavior of a titanium-indexed abutment used as a base for the installation of single ceramic crowns on Morse-taper implants. The average follow-up of the IA sets among the patients included in the study was 22.7 months, in which different clinical parameters were evaluated and their relationship with the stability of the marginal bone tissue. Our results showed an average bone remodeling around these IA sets of −0.67 ± 0.65 mm in mesial and −0.70 ± 0.63 mm in distal, corroborating other studies that evaluated implants in similar conditions and time [40,41,42]. Thus, bone loss around implants, regardless of the connection method, appears to be a physiological process.

It was possible to confirm the positive hypothesis of this study. The indexed abutment had acceptable behavior to support individual crowns. The index of the Morse-taper abutments brought some concerns about its clinical behavior. Some in vitro studies showed that it could decrease and/or interfere with the stability and performance of these abutments [20,28,29]. Within this regard, our study proved that in the Morse-taper indexed abutment model tested, the mechanical behavior was quite adequate, with no failures due to the loosening of these abutments found during the observation time of this study.

Regarding the rMBL of the IA sets evaluated separately by arch (maxilla and mandible), statistically, significant differences were found between both. In the mandible, the mean values were higher than those found in the maxilla. On this subject, several authors presented different results; some did not find differences between both arches [43], others reported greater bone remodeling in the maxilla [44,45], and others with higher values in the mandible, as found in our present study [46]. This last cited study related its results to the difference in implant insertion torque values, concluding that implants inserted with high torque values showed more increased bone remodeling than implants with low torque. Moreover, our group recently published a preclinical study that corroborates this correlation between the torque value and marginal bone remodeling [47]. However, in the present study, the insertion torque of the implants could not be evaluated due to the lack of this information in the patient’s clinical history.

Analyzing the implants placed in fresh sockets *versus* implants installed in healed sites, no statistically significant differences were found for marginal bone remodeling values at different periods, which agrees with the results of other authors [48]. Also, in the present study, the first analysis performed was after the installation of the definitive crown, and the initial bone remodeling of these sites (sockets) had already occurred. However, no differences were found in MBL values between implants installed in fresh sockets and healed places in radiographic analyses immediately after final crown placement, corroborating results reported in other studies [49,50,51]. As described in the Materials and Methods section, the waiting time to start rehabilitation for patients who received gap fillings was the same as for patients who received implants in healed sites since, according to Araujo et al., the fact that the implant occupies a large part of the socket decreases the amount of bone tissue to be formed in these sites [52,53].

Regarding immediate provisionalization vs. implants without immediate provisionalization, no statistical differences were found in marginal bone remodeling values. These results agree with Mangano et al.’s prospective multicenter study with two years of functional loading of 57 implants [54]. Still, Cooper et al., in their 5-year retrospective study, reported that peri-implant tissue parameters, which characterize implant success and contribute to the esthetics of implant rehabilitation, were similar in cases of immediate implant provisionalization when compared with healed ridges [55].

Among the main findings of our study, we can mention the differences found regarding the dimensions of the abutments used. As noted by other studies, abutment height is a key factor for the behavior and protection of the marginal bone of the implants [56,57,58]. These studies reported a higher marginal bone remodeling for abutments <2 mm compared to those ≥2 mm, corroborating the results obtained in the present study, which found statistically significant rMBL between the 2.5 and 3.5 mm in comparison to the abutment of 1.5 mm in transmucosal height. Other authors demonstrated that the biological width around an implant is 3–4 mm from the top of the peri-implant mucosa to the first bone-to-implant contact or the stabilized top of the adjacent bone [59]. Then, when the case needs to use abutments with a transmucosal height below 2 mm, the biological space is invaded, causing the organism to compensate for the lack of this space with bone remodeling [60].

Still referring to the dimensions of the abutments used, regarding the diameter of the abutments (3.5 or 4.5 mm), no significant differences were found in the final MBL values measurements. However, the abutments’ average and standard deviations were −0.61 ± 0.46 mm and −0.79 ± 0.71 mm (*p*-value = 0.1574) for abutments with 3.5- and 4.5-mm diameter, respectively. Although no statistically significant differences were found, the average of the measured values was higher for the Ø4.5 mm abutments, which corroborates the findings recently published in other studies showing that the smaller emergence angle of crown decreases the possibility of peri-implant tissues recession [61,62].

Finally, regarding the implants’ dimensions analyzed, the diameter (3.5 or 4.0 mm) did not cause a significant difference in the rMBL values, differing from the results obtained in another study carried out by our research group [63], which found a significant difference between the 2 implant diameters (MBL for Ø3.5 < 4.0 mm), and corroborating the results reported by other authors [64]. However, it must be considered that the number of implants with a diameter of 3.5 mm (*n* = 24) analyzed in our study was much smaller than that of 4.0 mm (*n* = 85). Borie et al., made some important considerations regarding the diameter of the implant used and the biomechanical behavior. They reported that implants with a larger diameter had better loading dissipation to the marginal bone tissue [65]. Regarding the length of the analyzed implants (9, 11, and 13 mm), a significant difference was found in the rMBL values between the 9 mm and 13 mm implants, corroborating the results reported by other authors [64,66].

Furthermore, a strong correlation was detected between rMBL and implant length. On the other hand, Mumcu et al., in a 36-month clinical study, showed no significant difference correlating the diameter and length of the implants [67]. However, there are many clinical publications on the implants’ behavior, and a great diversity of clinical situations and parameters analyzed makes it challenging to compare results with other studies.

There were limitations associated with this study. It was a retrospective study analyzing marginal bone in different sites (anterior and posterior) and maxilla and mandible, with varying densities of bone and impact masticatory forces. Moreover, there was a limited computational approach due to being a clinical study. We recommend future simulation in medical investigations using implants, similar to a recent publication [68], offering advantages, such as lower cost and faster results compared to clinical studies.

## 5. Conclusions

Within the limitations of this study, it was possible to conclude that better results (behavior and lesser marginal bone loss) were observed in the abutment with heights greater than 2.5 mm of the transmucosal portion and in implants with 13 mm length. Furthermore, this abutment showed a low incidence of failures within the period analyzed in our study. Thus, the longer the implant and the transmucosal portion (≥2.5 mm), the best performance may be achieved. These facts suggest, respectively, a greater BIC area and the mimetics of the supracrestal position of the soft tissue.

## Figures and Tables

**Table 1:** Mean, standard deviation, and statistical comparison between independent variables of the mesial and distal rMBL.

Independent Variables (Respectively)	Mean (±SD)	Mean (±SD)	*p*-Value
Mesial vs. distal rMBL (in mm)	(n = 109) −0.67 (0.65)	(n = 109) −0.70 (0.63)	0.5072
Mesial rMBL: maxilla vs. mandible sites (in mm)	(n = 58) −0.53 (0.48)	(n = 51) −0.83 (0.77)	0.0292 *
Distal rMBL: maxilla vs. mandible sites (in mm)	(n = 58) −0.57 (0.47)	(n = 51) −0.84 (0.75)	0.0192 *
Mesial rMBL: healed vs. socket sites (in mm)	(n = 74) −0.71 (0.68)	(n = 35) −0.84 (0.46)	0.1319
Distal rMBL: healed vs. socket sites (in mm)	(n = 74) −0.70 (0.69)	(n = 35) −0.69 (0.48)	0.7526
Mesial rMBL: immediate provisionalization vs. no provisionalization (in mm)	(n = 83) −0.71 (0.67)	(n = 26) −0.80 (0.45)	0.2224
Distal rMBL: immediate provisionalization vs. no provisionalization (in mm)	(n = 83) −0.62 (0.51)	(n = 26) −0.73 (0.49)	0.6650
Mesial rMBL: women vs. men (in mm)	(n = 70) −0.65 (0.62)	(n = 39) −0.68 (0.64)	0.5320
Distal rMBL: women vs. men (in mm)	(n = 70) −0.69 (0.65)	(n = 39) −0.71 (0.66)	0.3257

* Statistically significant difference. mm = millimeters.

**Table 2:** Data of the quantity, mean, standard deviation, and statistical analysis of each abutment used.

TMh Abutment	Quantity	Mesial rMBL	Distal rMBL	*t*-Test *p*-Value
1.5 mm	31 (28.4%)	−1.13 ± 0.39 mm	−1.15 ± 0.43 mm	0.9096
2.5 mm	50 (45.9%)	−0.62 ± 0.61 mm	−0.66 ± 0.60 mm	0.6649
3.5 mm	28 (25.7%)	−0.25 ± 0.64 mm	−0.26 ± 0.65 mm	0.3240
ANOVA *p*-value	---	<0.0001 *	<0.0001 *	---

TMh = Transmucosal height; * Statistically significant difference. mm = millimeters.

**Table 3:** Data of the quantity, mean, standard deviation, and statistical analysis of implant dimension were used.

Implant Dimensions	Quantity	Mesial rMBL	Distal rMBL	*t*-Test *p*-Value
Ø 3.5 mm	24	−0.55 ± 0.46 mm	−0.64 ± 0.55 mm	0.5619
Ø 4.0 mm	85	−0.74 ± 0.53 mm	−0.58 ± 0.60 mm	0.6420
*L* 9 mm	51	−0.79 ± 0.70 mm	−0.83 ± 0.74 mm	0.6796
*L* 11 mm	25	−0.66 ± 0.70 mm	−0.55 ± 0.50 mm	0.8226
*L* 13 mm	33	−0.48 ± 0.47 mm	−0.61 ± 0.49 mm	0.3487

Ø = Diameter; *L* = length. mm = millimeters.

## Data Availability

All data generated or analyzed during this study are included in this published article.
